# Time and Accuracy to Establish the Diagnosis of Soft Tissue Tumors: A Comparative Analysis from the Swiss Sarcoma Network

**DOI:** 10.1155/2022/7949549

**Published:** 2022-04-30

**Authors:** Hanna Wellauer, Gabriela Studer, Beata Bode-Lesniewska, Bruno Fuchs

**Affiliations:** ^1^Swiss Sarcoma Network, Luzern 6000, Switzerland; ^2^Department of Orthopaedics and Traumatology, Cantonal Hospital Winterthur, Winterthur 8401, Switzerland; ^3^Department of Radiation Oncology, Cantonal Hospital Lucerne, Lucerne 6004, Switzerland; ^4^Institute of Pathology Enge, Zurich 8005, Switzerland; ^5^Department of Plastic Surgery and Hand Surgery, University Hospital Zurich, Zurich 8091, Switzerland; ^6^Department of Orthopaedics and Traumatology, Cantonal Hospital Lucerne, Lucerne 6004, Switzerland

## Abstract

Soft tissue tumors are rare tumors, and their histological examination remains a challenge. The establishment of the correct initial histopathologic diagnosis is critical. However, due to the rarity of soft tissue and bone tumors and the inherent difficulty of their classification and diagnostics, discrepancies may occur in up to one third of cases. For these reasons, several studies recommend the involvement of experienced pathologists frequently performing sarcoma diagnostics. Until now, there is only scarce information about how long it takes to establish a correct sarcoma diagnosis. We thus analyzed all consecutive patients presented to the Swiss Sarcoma Network Tumor Board (SSN-MDT/SB) with a primary diagnosis of a soft tissue tumor over a 2-year period (01/2019 to 12/2020) based on a tumor biopsy. We then compared the final histopathological diagnosis of two comparable institutions with similar case load, but different workflows: (i) institution A, with an initial diagnosis performed by a local pathologist, and reviewed by a reference pathologist, and (ii) institution B, with the final diagnosis performed directly by a reference pathologist. In addition, we analyzed the time from biopsy to establishment of the diagnosis. A total of 347 cases were analyzed, 196 from institution A, and 149 from institution B. In 77.6% of the cases, the diagnosis from the local pathologist was concordant with the expert review. Minor discrepancies were found in 10.2% of the cases without any consecutive changes in treatment strategy. In the remaining 12.2% of the cases, there were major discrepancies which influenced the treatment strategy directly. Establishing the final report took significantly longer in institution A (4.7 working days) than in institution B (3.3 working days; *p* < 0.01). Our results confirm the importance of a pathological second review by a reference pathologist. We recommend direct analysis by experts, as diagnoses can be made more accurately and quickly. Within the SSN, establishing the sarcoma diagnosis is overall accurate and quick but still can be improved.

## 1. Introduction

Soft tissue tumors are rare tumors and histological examination remains a challenge [1]. The recently published WHO Classification of Soft Tissue and Bone Tumors [2] lists over 100 tumor entities including variants, often characterized by specific genetic aberrations, which can be detected by molecular diagnostic studies. Establishing the precise tissue diagnosis of a soft tissue or bone tumor is of utmost importance with respect to the choice of a correct treatment strategy for the patient. An incorrect histopathological diagnosis may lead to the initiation of an incorrect therapy with potentially severe or even lethal consequences for the patient [3–8].

Yet, due to the rarity of soft tissue and bone tumors and the inherent difficulty for a correct classification and diagnostic, discrepancies may occur in up to one third of cases [3–8]. For these reasons, several studies recommend the involvement of experienced pathologists who are involved in sarcoma diagnostics on a daily basis and who have access to auxiliary studies [3, 4, 9].

Various studies [4, 5, 7, 8] have shown that establishing the correct diagnosis for the treatment of soft tissue tumors is indeed a challenge, with 14% [10] to 43% [4] of all patients receiving an incorrect diagnosis, which could lead to incorrect treatment. Therefore, any multidisciplinary team (MDT) must assess these numbers constantly to compare with the reference benchmark for quality purposes.

Further, there is only scarce information on how long it takes to establish an expert review. Besides the correct diagnosis, the time from biopsy to establishing the diagnosis is an important quality indicator for the work-up of sarcoma patients. To the best of our knowledge, this factor has not yet been considered in published literature.

The patients treated in the Swiss Sarcoma Network (SSN) are either [1] referred directly to the member institutions prior to biopsy or [2] following a diagnosis of a mesenchymal tumor in an earlier outside biopsy. The current study concentrates on the first group in order to study the condition to optimize the diagnostic paths within the network. As the expansion of the network progresses in the future, there is hope that the percentage of the tissue studies outside the network (including “whoops” unintended resections) will diminish. Herein, we report first on the quality of accuracy in establishing the sarcoma diagnosis within the Swiss Sarcoma Network, and second, assess how long it takes to establish the diagnosis including expert review analysis.

## 2. Materials and Methods

All consecutive patients presented at the Swiss Sarcoma Network Board with a primary diagnosis of a soft tissue tumor from January 1, 2019, to December 31, 2020, were included in this study. Patients with incomplete records were excluded. A record was marked as incomplete when, for example, a case from institution A was missing an expert review, or when a case from institution B was initially diagnosed locally. The diagnoses were classified according to the WHO into benign, intermediate and malignant [11].

**Figure 1:**
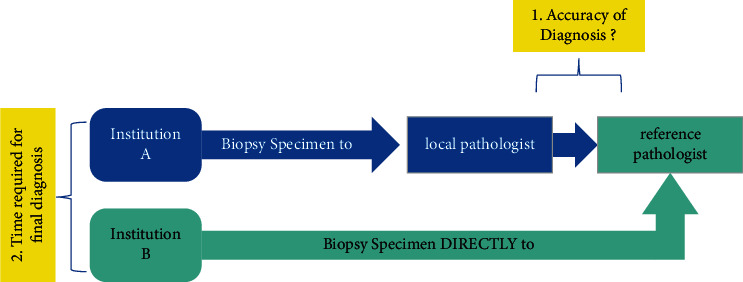
Workflow of the study.

The biopsies of the two institutions were analyzed and compared. The samples of institution A were initially analyzed by the local pathology institute. This is a general pathology institute without specific subspecialization. Afterwards, the samples being reviewed and assessed by a reference institute pathologist specialized in soft tissue tumors. Conversely, institution B cases were assessed directly by the reference institute pathologist. These workflows are illustrated in Figure 1.

To determine the time from biopsy to the establishment of the diagnosis, the days between the arrival of the tissue specimen at the pathology institute until the date of the final report were calculated. Weekend days or holidays were not counted, unless the report was issued on one of these days. In the analysis of the current study only cases which can be diagnosed by conventional histopathologic staining, immunohistochemistry, and FISH were included, as these studies have a short turn-around-time of one to two days. The cases requiring PCR or NGS based analyses were excluded as they methodically require several days independently of the performance of the pathologist.

The accuracy of the diagnoses of the local histopathology institute A and the expert analysis was examined in a second step. Here, the diagnoses of institute A were compared with the expert opinion and divided into 3 groups according to the classification of Thway et al. [6]:

- Cases without diagnostic discrepancy between local and reference institutions were classified into category A
- Category B includes cases with minor discrepancy in diagnosis but without therapeutic consequences
- Category C contains all cases where the diagnosis from the reference pathologist changed the treatment

In addition, all cases where the final report from institution A did not establish a diagnosis were consequently classified under category C [6].

The data were collected using the Adjumed ® -Database (www.adjumed.ch; Zurich, Switzerland) and analyzed with the statistical package “stats” of the open source software “R” [12].

The cantonal ethic commission has approved the application of the Swiss Sarcoma Network under the agreement number BASEC-NR 2019-01107. The study is also registered on https://climincaltrials.gov with the number NCT04300257 [13].

## 3. Results

### 3.1. Patient and Tumor Characteristics

A total of 347 cases were analyzed, 196 from Institution A and 149 from Institution B. 179 patients were female and 168 were male, and the median age was 55 (range 12–90) years. 163 cases were classified as benign (46.9%), 114 cases were malignant (32.8%), 66 cases were intermediate (19%), and 4 cases were unclassifiable (see Table 1).

The most common benign diagnosis was lipoma (69 cases, 42.3% of all benign tumors), followed by Schwannoma (11 cases, 6.7%). Regarding the malignant diagnosis, undifferentiated/unclassified sarcoma was the most common diagnosis (19 cases, 16.6% of all malignant tumors) followed by the dedifferentiated liposarcoma (14 cases, 12.2%, see Table 2).

### 3.2. Accuracy

Of the 196 tumors specimens from institution A (which underwent initial diagnosis by a local pathologist followed by specimen being reviewed by a reference pathologist, see Figure 1), 152 tumors (77.6%) were diagnostically concordant according to category A. Of the latter 152 tumors, 46.7% were benign, 18.4% were intermediate, 33.5% were malignant, and 1.4% unclassifiable. There were 20 cases (10.2%) with minor discrepancies, according to category B (Table 3). Of these, 70% were malignant, 15% intermediate, and 15% benign diagnoses. There were 24 tumors (12.2%) with major diagnostic discrepancies (Table 3) according to category C. 50% of these were malignant cases. From these major discrepancies, 12 cases were classified in this category because of a missing diagnosis in the final report from institution A. In one case, there was a reclassification from benign to malignant and one case was reclassified from malignant to benign. A summary of all original diagnoses, which were discordant from the expert review is shown in Table 3.

### 3.3. Analysis of Time to Diagnosis

**Figure 2:**
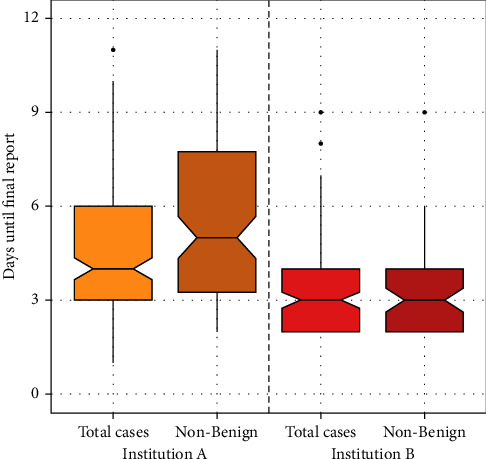
Days until final report.

Establishing the final report took on average 4.7 working days for institution A, which is significantly longer than the 3.3 days required by institution B (Figure 2). 10 cases were excluded from the analysis (7 from institution A and 3 from institution B) due to the necessity of NGS for the final diagnosis. We analyzed the data with a two-sided Wilcoxon *t*-test and found a *p* value of *p* < 0.01.

If only malignant diagnoses were considered for analysis, establishing the diagnosis averaged 5.2 days in institution A, and 3.4 days, respectively, for institution B (*p* < 0.01, see Figure 2).

According to the most commonly diagnosed lesion of all, the diagnosis of a lipoma averaged 4.6 days at institution A and 3.2 days, respectively, at institution B (*p* < 0.01). Accordingly, and with respect to undifferentiated/unclassified sarcoma, institution A required 5.2 days, and institution B 3.0 days (*p* < 0.01).

## 4. Discussion

To the best of our knowledge, this is the first analysis comparing the duration of a histological review to establish a sarcoma diagnosis. Our results confirm the importance of a second pathological review by a reference pathologist. With an overall concordance of 77%, the results are comparable to the already published literature.

In 1986, Presant et al. [7] first reported on a histopathologic peer review of specimens from 216 consecutive patients with soft-tissue or bone sarcomas by a panel of three pathologists. Subtype of sarcoma, degree of confidence in diagnosis, and grade were compared with agreement or disagreement in pathologic opinion from the primary member institution versus the pathology review panel. There was a complete agreement between the primary pathologist and reviewer in 66% of cases. However, after the review, 12 cases (6%) were considered not to be sarcoma. In 27% of cases, the subtype of sarcoma was felt to be incorrect by reviewers.

In 2008, Lehnhardt et al. [5] reviewed 603 patients who were operated with the diagnosis of soft tissue sarcoma. They found a concordance in primary diagnostics of 28.3% for pathologists in private clinics, 29.6% for hospital affiliated pathologists, 36.8% for academic medical centers, and 70.5% for the department of pathology at their institution.

In 2010, Lurkin et al. [8] analyzed all histological data of all patients diagnosed with sarcoma in the Rhone-Alpes region between March 2005 and February 2006. Primary diagnoses were systematically compared with second opinions from regional and national experts. They included 366 patients; of these, 199 (54%) had full concordance between primary diagnosis and second opinion, 97 (27%) had partial concordance (identical diagnosis), and 70 (19%) had complete discordance.

Ray-Coquard et al. [4] reviewed the histological data of patients diagnosed with sarcoma in Rhone-Alpes (France), Veneto (Italy) and Aquitaine (France) over a 2-year period. Initial diagnoses were systematically compared with the second opinions from members of the group of pathologists of the GSF-GETO (French Unicancer Sarcoma Group). 1463 cases matched the inclusion criteria and were analyzed. Full concordance between primary and second diagnosis was observed in 824 (56%) cases, partial concordance in 518 (35%) cases and complete discordance in 121 (8%) cases.

A summary of the studies can be found in Table 4.

Interestingly, and specifically contrasting the analysis between benign and malignant lesions, the uncertainty to establish the correct diagnosis was greater in malignant lesions. Considering the analysis of minor discrepancies in the diagnosis comparing first line with expert review, the expert review delivers more diagnostic details or a supplement in the classification without obvious consequences regarding the treatment modality, specifically also for malignant diagnoses.

Our study has several limitations: The number of biopsies analyzed is still relatively small, and many diagnoses are benign, thereby not allowing further subgroup analysis. Also, considering the rarity of the disease and the 68 sarcoma entities included therein, further subtype analysis is not possible. The definition of diagnostic discordances is not always obvious and may skew the results. Arbitrarily, descriptive pathology reports without specification of dignity were classified as major discrepancies because adequate treatment can only be initiated when the final diagnosis is made.

Although there is a significant difference in the time to diagnosis, one may critically question to what extent this value has an influence on the time to diagnosis and further therapy. The time it takes to establish the histological examination is only one step on this path. It would therefore be interesting if a further study examines not only the duration of the biopsy, but the entire process from the suspected diagnosis to the initiation of the correct therapy. But from the point of view of the patient who must wait for a diagnosis, every day that is gained with a faster diagnosis is worth a lot. In addition, a rapid histological diagnosis is essential for a timely discussion at the multidisciplinary sarcoma board.

Any additional examination, especially if not done in the same institution, will lead to delays in the diagnostic process.

Several studies confirmed that a centralized pathological review improved the quality of the diagnosis. Lurkin et al. [8] support the direct analysis by an expert pathologist because of the multitude and complexity of sarcoma tumors. Also, the access to molecular biology analysis can be provided. Compared to the recommendation of the ECCO Essential Requirements for Quality Cancer Care, the pathway of Institution B is to be favored [14].

In a small country like Switzerland, and with sarcoma being a rare disease, establishing the correct pathological diagnosis is very challenging. The main reason is the small amount of cases per individual hospital. Compared with the volume of international sarcoma reference centers, the data of the entire country needs to be pooled and shared to reach high enough numbers for expert experience and teaching purposes. With the recently established Swiss Sarcoma Network, allowing real-world outcome analytics, there is the possibility to improve the precision, timeliness, and accuracy of sarcoma diagnosis in Switzerland in the near future. As of now, 7 central referral institutions joined the Swiss Sarcoma Network so far and benefit from a second opinion by an expert pathologist.

There is no clear definition in the literature on how a sarcoma expert is defined. As for the pathologists, the sarcoma experts within the Swiss Sarcoma Network are defined by their specific training, their specific sarcoma interest, defined by dedication of >30–50% of their duty time spent on treating sarcoma patients, their yearly scientific contributions, their number of cases reviewed and/or treated per year, and their participation of the weekly multidisciplinary tumor board including the number of discussed cases and strategic decisions.

## 5. Conclusions

The diagnosis of sarcoma remains challenging. According to our study and the current literature, an expert review by an experienced pathologist within a network such as the Swiss Sarcoma Network proves to be highly useful and beneficial for the patient both regarding accuracy and timeliness to establish the diagnosis. Establishing the sarcoma diagnosis as early as possible after biopsy is a critical quality indicator for a multidisciplinary team. Considering the rapidly rising health care costs, the potential increase in cost efficiency of such a process needs to be determined next.

## Figures and Tables

**Table 1:** Patient and tumor characteristics.

Total	347

Institution A	198 (57%)
Institution B	149 (43%)
Male	168 (48.5%)
Female	179 (51.5%)
Median age	55 (range 12–90) years
Benign diagnosis	163 (46.9%)
Intermediate diagnosis	66 (19%)
Malignant diagnosis	114 (32.8%)
Unclassified diagnosis	4 (1.2%)

**Table 2:** Most common diagnoses.

Benign	Intermediate	Malignant
Lipoma (69 cases)	Atypical lipomatous tumor/well differentiated liposarcoma (17 cases)	Unclassified/undifferentiated sarcoma (19 cases)
Schwannoma (11 cases)	Aneurysmal bone cyst (11 cases)	Dedifferentiated liposarcoma (14 cases)
Intramuscular myxoma (8 cases)	Desmoid‐type fibromatosis (9 cases)	Leiomyosarcoma (11 cases)

**Table 3:** Minor/major discrepancies.

Minor discrepancies		
*Benign*		
	*Institution A*	*Institution B*

L107	Fibroblastic/myofibroblastic proliferates in predominantly tight connective tissue with partly regressive changes.	Collagen-rich myofibroblastic proliferation
L108	Chondrogenic neoplasm, highly differentiated	Enchondroma
L198	Fibrin and blood, intercalated with some lamellar bone tissue and connective tissue	Intraosseous ganglion
*Intermediate*		
	*Institution A*	*Institution B*
L31	Spindle-cell, partly multinucleated giant-cell tumor with osteoid formation	Aneurysmal bone cyst
L34	Giant cell tumor of the soft tissue	Plexiform fibrohistiocytic tumour
L112	Chondroid neoplasia with cancellous bone	Epiphyseal atypic chondrogenic tumor
*Malignant*		
	*Institution A*	*Institution B*
L4	Spindle-cell high-grade sarcoma	Spindle and pleomorphic high-grade malignant unclassified sarcoma G3
L11	Spindle-cell pleomorphic sarcoma, high grade, with evidence of myogenic differentiation	Leiomyosarcoma
L19	Epithelioid sarcoma (proximal type)	Epithelioid angiosarcoma
L29	Lymph node metastasis of a solid tumor (differential diagnosis: clear cell sarcoma or malignant melanoma)	Lymph node metastasis of malignant melanoma
L35	Pleomorphic undifferentiated sarcoma with necrosis zones	Pleomorphic liposarcoma (G3)
L60	Spindle-cell pleomorphic neoplasia with striated muscles	Sclerosing epithelioid fibrosarcoma
L63	Sarcoma, spinel and partly pleomorphic cells	Spindle and pleomorphic cell soft tissue sarcoma at least G2 with FNCLCC score of 4
L64	Highly differentiated liposarcoma	Dedifferentiated liposarcoma with low-grade dedifferentiated portion, malignancy grade at least G2
L84	Myxofibrosarcoma	Undifferentiated spindle cell sarcoma
L110	Myxofibrosarcoma (high grade)	High-grade, unclassifiable spindle cell sarcoma (G2)
L140	Undifferenciated pleomorphic sarcoma	High-grade, unclassifiable spindle cell sarcoma (G2)
L157	Myxofibrosarcoma, high grade	High-grade, unclassifiable spindle cell sarcoma (G2)
L188	Pleomorphic highly proliferative tumor	Giant cell-rich leiomyosarcoma at least G2
L201	Myxofibrosarcoma (high grade)	Spindle cell sarcoma at least G2

*Major discrepancies*		
*Benign*		
	Institution A	Institution B

L2	Fat necrosis	PHAT (pleomorphic hyalinizing angiectatic tumor of soft parts)
L37	Fibrin-rich connective tissue with low chronic inflammation and regressive changes	Nodular fasciitis
L52	Mature teratoma/dermoid	Spinal dermoid cyst
L57	Spindle-cell mesenchymal myofibroblastic proliferation with low MIB-1 proliferation rate along with skeletal muscles	Intramuscular myxoma
L66	Parts of a spindle-cell myxoid-chondroid impinging neoplasia	Benign portion of a peripheral nerve sheath tumor
L109	Slightly atypical spindle cell tumor with myxoid background and increased proliferation (Ki67) of approx. 30%.	Myofibroblastic proliferation of the nodular fasciitis type
L113	Low-grade fibromyxoid sarcoma	Intramuscular myxoma
L145	Smooth-muscular proliferation with scaly calcifications as well as circumscribed ossification without necrosis or evidence of mitoses	Leiomyoma of the deep somatic soft tissues
L195	Intramuscular lipoma	Intramuscular haemangioma
*Intermediate*		
	*Institution A*	*Institution B*
L100	Spindle cell mast cell-rich proliferation with low proliferation rate and immunohistochemically S-100 positive with negativity for SOX-10	Solitary fibrous tumor (SFT)
L101	Plump spindle-cell tumour with multiple multinucleated giant cells	Periosteal aneurysmal bone cyst (ABC)
L117	Cell-rich neoplasia of oval, plump spindle mononuclear cells intermixed with giant cells and haemorrhage residues in connective tissue.	Tenosynovial giant cell tumor of the diffuse type
*Malignant*		
	*Institution A*	*Institution B*
L1	Epithelioid sarcoma	Angiosarcoma
L6	Osteosarcoma	Chondrosarcoma
L7	Highly differentiated/dedifferentiated or a myxoid liposarcoma	Dedifferentiated liposarcoma (low grade)
L51	Myxoid chondrosarcoma	Myxoid liposarcoma (G1)
L70	Chondroid and focal spindle cell neoplasia	Mesenchymal chondrosarcoma
L76	Slightly hypercellular chondrogenic tissue, connective tissue and skeletal muscle	Conventional chondrosarcoma
L94	Pleomorphic liposarcoma	Round cell liposarcoma G3
L150	Small blue round cell tumor with low proliferation (Ki67) of approx. 10-15%.	Granulosa cell tumor
L164	Atypical lipomatous tumor/well-differentiated liposarcoma	Dedifferentiated liposarcoma, at least G2
L171	Neoplasia, predominantly spindle cell in cancellous bone with focal evidence of irregular osteoid.	Osteosarcoma, high grade
L191	Infiltrates of small, round and blue cell neoplasia	Poorly differentiated neuroendocrine carcinoma (Merkel cell carcinoma)
L204	Spindle and pleomorphic cell neoplasm with myxoid background of partial expression of MDM2	High-grade myxofibrosarcoma (G2-3)

**Table 4:** Overview of literature.

Author (year)	Interval	No. of cases	% concordance	% minor deviations	% major deviations
Peasant (1986)	May 1974 to May 1982	216	66% (137)		
Lehnhardt (2008)	1995 to 2001	603	28.3–70.5%		
Lurkin (2010)	March 2005 to February 2006	366	54%(199)	27% (97)	19% (70)
Ray-Coquard (2012)	March 2005 to February 2007 resp. January 2007 to December 2008	2016	56% (824)	35% (518)	8% (121)
Our study	January 2019 to December 2020	196	77.6% (152)	10.2% (20)	12.2% (24)

## Data Availability

The data used to support the findings of this study may be released from the Adjumed®-Database upon request to the Swiss Sarcoma Network (office@sarcoma.surgery).
